# The role of refugee status and mental disorders regarding subsequent labour market marginalisation: a register study from Sweden

**DOI:** 10.1007/s00127-020-01842-8

**Published:** 2020-02-13

**Authors:** Thomas Niederkrotenthaler, Ellenor Mittendorfer-Rutz, Fredrik Saboonchi, Magnus Helgesson

**Affiliations:** 1 Unit Suicide Research & Mental Health Promotion, Department of Social and Preventive Medicine, Centre for Public Health, Medical University of Vienna, Kinderspitalgasse 15, 1090 Vienna, Austria; 2 Division of Insurance Medicine, Department of Clinical Neuroscience, Karolinska Institutet, 171 77 Stockholm, Sweden; 3 Red Cross University College, Stockholm, Sweden

**Keywords:** Migration, Labour, Disability pension, Unemployment, Mental disorders, Sweden

## Abstract

**Purpose:**

This study aimed to assess the role of refugee status and specific mental disorders regarding subsequent labour market marginalisation.

**Methods:**

Prospective cohort study of all refugees (*n* = 216,930) and Swedish-borns (*n* = 3,841,788), aged 19–60 years, and resident in Sweden in 2009. Hazard ratios (HRs) with 95% Confidence Intervals (CIs)  for long-term unemployment (> 180 days) and disability pension (DP) were calculated with Cox regression analyses.

**Results:**

Mental disorders were more prevalent in refugees compared to Swedish-born individuals, with greatest differences seen for post-traumatic stress disorder (PTSD; refugees 1.3%; Swedish-born individuals 0.1%). Regarding long-term unemployment, refugees without a mental disorder had an adjusted HR (aHR) of 2.68 (95% CI 2.65–2.71) compared to Swedish-born individuals without mental disorders, which was above the aHR of refugees (aHR 2.33, 95% CI 2.29–2.38) and Swedish-born individuals (aHR 1.44, 95% CI 1.43–1.45) with mental disorders. Regarding DP, compared to Swedish-born individuals without mental disorders, the aHRs were 1.44 (95% CI 1.34–1.54) for refugees without, but 6.11 (95% CI 5.84–6.39) for refugees with mental disorders. Swedish-born individuals with mental disorder had an aHR of 3.96 (95% CI 3.85–4.07). With regard to specific disorders, the aHRs for refugees, as compared to Swedish-born individuals without mental disorders, were markedly increased for all disorders (e.g. PTSD: long-term unemployment aHR: 2.03 (95% CI 1.89–2.18); DP 7.07 (95% CI 6.42–7.78).

**Conclusion:**

Mental disorders are more prevalent in refugees than in Swedish-born individuals but do not appear to increase their risk of long-term unemployment. Refugee status and mental disorders strongly contribute to the risk of DP, indicating that factors beyond medical considerations contribute to their granting of DP.

## Introduction

Labour market marginalisation, i.e. severe problems in finding and keeping a job, is a growing and serious public health and economic problem in Europe [1]. Migrants have in general a weaker attachment to the labour market compared to Swedish-born individuals [2], and particularly non-Western migrants have been shown to have a lower educational level compared to both Western migrants and Swedish-born individuals, which contributes to marginalization on the labour market [3]. A group that has received little attention in research regarding labour market marginalization are refugees, who generally have higher risks of both, unemployment and disability pension [4].

Refugees are reported to have a higher prevalence of mental disorders compared to both Swedish-born individuals and other migrant groups [5]. Their increased risk for mental disorders is often ascribed to traumatic events both before and during the migration process, but also to living conditions including lack of labour market integration after resettlement in the host country [6]. Consequently, mental disorders may act as both predictors and consequences of marginalization [7, 8]. Previous research on labour market marginalization among young individuals up to 35 years in Sweden revealed that non-Western immigrants with mental disorders had a higher risk of unemployment, but a lower risk of disability pension as compared to Swedish-born individuals with such disorders [9]. It remains unclear if these patterns are consistent for refugees, and how mental disorders modify the effect of refugee status on different markers of labour market marginalisation, i.e. not only unemployment but also disability pension.

According to previous research the risk of labour market marginalization, particularly disability pension, varies considerably for different mental disorders, with schizophrenia, personality disorders and mental retardation carrying the highest risk [10]. Marginalization patterns vary depending on specific markers used [9, 10], and a sole focus on unemployment is not sufficient to describe the effects of mental disorders on related outcomes [10]. In spite of the higher prevalence of mental disorders in refugees compared to host populations, and differences in diagnostic patterns, there are so far no studies available that have analysed the role of specific mental disorders in refugees with regard to the subsequent risk of labour market marginalisation.

## Aim

The study aim was to investigate to what extent refugee status modifies the association between mental disorders and subsequent labour market marginalisation, measured as both long-term unemployment and disability pension.

## Methods

### Study population

This population-based prospective cohort study consisted of all individuals 19–60 years who were resident in Sweden (*n* = 5,092,354) on 31st of December 2009. Individuals on disability pension during 2009 (*n* = 372,046), with missing data on reason for settlement to Sweden (*n* = 262,579), with a reason for migration other than being a refugee (*n* = 383,095) and persons with ambiguous reasons for migration (*n* = 15,916) were excluded. The final study population consisted of 4,058,718 individuals, whereof 216,930 (5.3%) individuals were refugees.

### Register data

People were identified through registers from Statistics Sweden and data were available for each individual retrospectively and followed-up prospectively up to 31st December 2013 based on information from the following three agencies: (1) Statistics Sweden: *LISA (Longitudinal integration database for health insurance and labour market studies)*; information on socio-demographic factors measured on December 31, 2009: age, sex, educational level, family situation, type of living area (small cities/villages, medium and large cities), date of immigration, labour market attachment and unemployment; *STATIV (Longitudinal database for integration studies*): Information on reason for settlement in Sweden 1997–2013; (2) Social Insurance Agency: information on sickness absence and disability pension (DP) (date) 2009–13; and (3) the National Board of Health and Welfare: *National patient register*: date and diagnosis of specialised psychiatric and somatic in- and outpatient care starting from 2006 to 2009; *Cause of death register*: date and cause of death from 1960 and onwards and *National prescribed drug register*: prescription of dispensed psychiatric medication: date of prescription and dispensing, from 2006 to 2009.

### Refugee status and categorization of region of birth

Refugees were defined as migrants who came to Sweden as a refugee, due to humanitarian reasons or need of protection. Swedish-born individuals were used as the comparison group.

### Outcome measures

The cohort was followed 2010–13 with regard to long-term unemployment, defined as > 180 annual days registered as full-time unemployed at the Swedish Public Employment Service during the follow-up period, and newly granted disability pension.

### The Swedish social insurance system

In Sweden, the insurance system consists of a basic and a voluntary insurance. The voluntary insurance is related to income from work while the basic insurance is granted to every resident from 20 years of age, who is registered at the employment agency and has a job-seeking plan. Also students are eligible. Individuals above 30 years with impaired capacity to work due to disease or injury can be granted permanent disability pension. Individuals between 19–29 years of age can receive temporary disability pension due to disease or injury but can additionally also receive disability pension due to incapacity to complete compulsory/upper secondary school [3].

### Covariates

Covariates in the analyses were the following: (I) *Sociodemographic factors*: sex (male and females), age (19–24 years, 25–34 years, 35–44 years, 45–54 years and 55–60 years), educational level [high (> 12 years), medium (10–12 years) and low (< 10 years)], type of living area (big cities, medium sized cities and small cities/villages), and family situation (married/cohabiting with children at home, married/cohabiting with no children at home, single with children at home, single with no children at home, and adolescents up to 20 years of age at home) (all variables were measured at 31st December 2009);

(II) *Work-related factors:* Labour market attachment, measured as income during 2009 categorised as (a) income from work, (b) income, but not from work and (c) no income; unemployment in 2009 (no unemployment (reference category), 1–180 days and > 180 days), sickness absence in 2009 (no sickness absence (reference category), 1–90 days and > 90 days).

(III) *Health factors:* a dichotomised annual variable for a record of a main or side diagnosis from in- or specialised outpatient health care due to somatic disorders during 2006–2009 (International Classification of Disorders version 10, ICD-10: A00-E99, G00-T99) and a dichotomised variable for mental disorders, measured as a record of a main or side diagnosis from in- or specialised outpatient health care due to mental disorders 2006–2009 (ICD-10: F00—F99) or as prescription of anxiolytics, sedatives and antidepressants 2006–2009, coded according to the Anatomical Therapeutic Chemical Classification (ATC) codes N05B, N05C and N06A, respectively. In addition, we categorized specific mental disorders based on the ICD-10 diagnoses into depressive disorders (F32, F33), bipolar disorder (F31), anxiety disorders (F40, F41, F42), post-traumatic stress disorder (PTSD; F43.1), other stress-related disorders (F43.0, F43.2, F43.8, F43.9), and other mental disorders (other F-diagnoses).

### Statistics

We performed Cox proportional-hazard regression models. Crude and adjusted hazard ratios (HRs) of long-term unemployment and disability pension with 95% confidence intervals (CIs) were calculated for refugees with and without mental disorders where Swedish-born individuals without mental disorders were the reference category. Interaction tests of refugee status and presence of any mental disorder were significant (unemployment *p* < 0.001; disability pension *p* < 0.001). We also assessed associations of specific mental disorders among Swedish-born individuals and refugees with long-term unemployment and disability pension, with Swedish-born individuals without mental disorders as the reference group. Censoring was due to emigration, death or end of follow-up, whichever came first. In the analyses of unemployment, we censored also for disability pension. Missing values were coded as separate categories. Adjustment was made in steps for sociodemographic, work-related and health- related factors (see above, “covariates”, for specific variables in these categories). All analyses were conducted by SPSS Statistical Software, version 23.

### Ethics statement

This project was evaluated and approved by the regional ethical review board in Stockholm, Sweden and was conducted in accordance with the Declaration of Helsinki.

## Results

### Descriptive information

As compared to Swedish-born individuals, refugees were more often males (58.7% vs. 52.1%), younger, less educated (low educational level: 24.2% vs. 10.6%) more often living with children, and more often resident in large cities (48.1% vs. 36.4%). Refugees had much more frequently income from other sources than work than Swedish-born individuals (44.7% vs. 15.8%), and were more often long-term unemployed (10.4% vs. 2.4%) or on long-term sick leave (2.7% vs 2.0%; see Table 1) at baseline.

**Table 1:** Descriptive statistics of all refugees (*n* = 216,930) and Swedish-born individuals (*n* = 3,841,788) 19–60 years of age, who were resident in Sweden on December 31 2009 and followed up from 2010 to 2013

Variable	Refugees *n* (%)	Swedish-born individuals, *n* (%)
Sex		
Male	127,288 (58.7)	2,001,537 (52.1)
Female	89,642 (41.3)	1,840,251 (47.9)
Age, years		
19–24	34,395 (15.9)	604,739 (15.7)
25–34	56 409 (26.0)	862,948 (22.5)
35–44	58,252 (26.9)	994,786 (25.9)
45–54	53,844 (24.8)	885,628 (23.1)
55–60	14,030 (6.5)	493,687 (12.9)
Educational level, years		
0–9	52,409 (24.2)	408,865 (10.6)
10–12	88,901 (41.0)	1,979,987 (51.5)
> 12	66,534 (30.7)	1,447,559 (37.7)
Missing	9086 (4.2)	5377 (0.1)
Family situation*		
Married without	16,896 (7.8)	389,201 (10.1)
Married with	98,028 (45.2)	1,516,424 (39.5)
Single without	76,623 (35.3)	1,508,914 (39.3)
Single with	18,370 (8.5)	256,509 (6.7)
Living with parents, ≤ 20 years	7011 (3.2)	170,738 (4.4)
Missing	2 (0.0)	2 (0.0)
Type of living area		
Big cities	104,415 (48.1)	1,397,354 (36.4)
Medium-sized cities	78,942 (36.4)	1,383,519 (36.0)
Small cities/villages	33,573 (15.5)	1,060,915 (27.6)
Labour market attachment		
Income from work	119,973 (55.3)	3,233,169 (84.2)
No income from work	96,957 (44.7)	608,619 (15.8)
Unemployment		
No	154,953 (71.4)	3,398,534 (88.5)
1–180 days	39,509 (18.2)	350,204 (9.1)
> 180 days	22,468 (10.4)	93,050 (2.4)
Sickness absence		
No	198,517 (91.5)	3,510,985 (91.4)
1–90 days	12,609 (5.8)	253,869 (6.6)
> 90 days	5804 (2.7)	76,934 (2.0)

^*^With/without children living at home

Specific mental disorders were more prevalent in refugees than among Swedish-borns; particularly PTSD (1.3% vs. 0.1%); other stress-related disorders (1.9% vs. 0.9%); depression (3.0% vs. 1.9%); and anxiety-related disorders (2.4% vs. 1.9%; Table 2).

**Table 2:** Prevalence of specific mental disorders*, diagnosed from inpatient or spezialized outpatient care between 2006 and 2009 in all refugees (*n* = 216,930) and Swedish-born individuals (*n* = 3,841,788) 19–60 years who were resident in Sweden on 31st of December 2009

Variable	Refugees	Swedish-borns
No mental disorder	200,273 (92.3)	3,636,824 (94.7)
Depressive disorders	6462 (3.0)	73,989 (1.9)
Bipolar disorder	375 (0.2)	11,727 (0.3)
Anxiety disorders	5249 (2.4)	72,507 (1.9)
Post-traumatic stress disorder (PTSD)	2924 (1.3)	2801 (0.1)
Other stress-related disorders	4104 (1.9)	36,006 (0.9)
Other mental disorders	5554 (2.6)	97,642 (2.5)
Somatic disorders (any)	135,071 (62.3)	2,235,570 (58.2)

^*^ International Classification of Diseases 10th Revision ICD-10 Codes: depressive disorders: F32, F33; bipolar disorder: F31; anxiety disorders: F40, F41, F42; post-traumatic stress disorder (PTSD): F43.1; other stress-related disorders: F43.0, F43.2, F43.8, F43.9; other mental disorders: other F-diagnoses; somatic disorders: A00-E99, G00-T99

### Risk of long-term unemployment and disability pension

Refugees had a HR of 4.67 for long-term unemployment in the crude analysis (data not shown). This ratio diminished to 1.80 after adjustment for socio-economic variables, somatic and mental disorders at baseline. Regarding disability pension, refugees had a HR of 2.29 in the crude analysis. This HR was reduced to 1.61 after adjustment.

As compared to Swedish-born individuals without a mental disorder, the risk of long-term unemployment of refugees turned out to be considerably higher and different from Swedish-borns. Compared to Swedish-borns without mental disorders, mental disorders did not add to this marginalization in refugees, but was associated with a lower aHR (refugees without mental disorders: aHRs 2.68 vs. 2.33 in refugees with mental disorders; Table 3).

**Table 3:** Interaction between mental disorders as diagnosed in inpatient and specialized outpatient care from 206 to 2009 and refugee status for long-term unemployment (> 180 days) and disability pension between 2010 and 2013 for all native Swedes (*n* = 3,841,788) and refugees (*n* = 216,930) 19–60 years who were resident in Sweden at 31st of December 2009

Variable	Event *N* (rate per 100,000 per year)	Crude HR (95% CI)	Model 1 HR (95% CI)
Long-term unemployment (> 180 days)			
Swedish-born without mental disorder	144,659 (1153.2)	1 (Ref)	1 (Ref)
Swedish-born with mental disorder	66,771 (2365.5)	2.11 (2.09, 2.13)	1.44 (1.43, 1.45)
Refugee without mental disorder	39,120 (5854.1)	5.57 (5.51, 5.63)	2.68 (2.65, 2.71)
Refugee with mental disorder	12,283 (6157.6)	5.97 (5.86, 6.08)	2.33 (2.29, 2.38)
Disability pension			
Swedish-born without mental disorder	9538 (76.0)	1 (Ref)	1 (Ref)
Swedish-born with mental disorder	20,282 (718.5)	9.61 (9.38, 9.84)	3.96 (3.85, 4.07)
Refugee without mental disorder	973 (145.6)	1.93 (1.81, 2.07)	1.44 (1.34, 1.54)
Refugee with mental disorder	2831 (1419.2)	19.31 (18.51, 20.13)	6.11 (5.84, 6.39)

Model 1 adjusted for sex, age, educational level, type of living area, family situation, labour market attachment, unemployment at baseline, sickness absence at baseline, somatic hospitalization or specialized outpatient care 2006–2009

*CI* confidence interval, *HR* hazard ratio, *Ref* reference category

In contrast, mental disorders affected the subsequent risk of disability pension of Swedish-borns and refugees in similar ways, but refugee status alone was related to a nearly doubled risk of disability pension (HR 1.93) in the crude analysis, which was reduced to 1.44 in the adjusted analysis. This resulted in an extraordinarily high risk of this type of marginalization in the group of refugees with mental disorder versus Swedish-borns with mental disorders (aHR 6.11 vs. 3.96; Table 3). In the unadjusted analyses, the risk of long-term unemployment among refugees, and—even more pronounced—of disability pension was tremendously increased compared to Swedish-borns, reaching up to a nearly 20-fold risk of disability pension (HR 19.31) in refugees with mental disorders. The adjustment of socio-demographic variables, particularly educational level, reduced these risks markedly (Table 3).

With regard to the risk of long-term unemployment and disability pension due to specific mental disorders (Table 4), the risk for refugees with the respective disorders was markedly increased as compared to Swedish-born individuals without mental disorders. This was seen across the whole spectrum of disorders, and risks were considerably higher among refugees than among Swedish-born with the respective disorder (Table 3). Adjustment for socio-demographic and health-related variables resulted in an attenuation of estimates.

**Table 4:** Hazard ratios and 95% Confidence Intervals for long-term unemployment (> 180 days) and disability pension between 2010 and 2013, for specific mental disorders (diagnosed between 2006 and 2009) for all Swedish-born individuals (*n* = 3,841,788) and refugees (*n* = 216 930), 19–60 years, who were resident in Sweden on 31st of December 2009

Type of mental disorder	Long-term unemployment		Disability pension	
	Crude HR (95% CI)	Model 1 HR (95% CI)	Crude HR (95% CI)	Model 1 HR (95% CI)
All specific disorders				
Swedish-born without mental disorder	1 (Ref)	1 (Ref)	1 (Ref)	1 (Ref)
Refugees without mental disorders	5.10 (5.05, 5.15)	2.46 (2.44, 2.49)	2.26 (2.16, 2.36)	1.67 (1.59, 1.74)
Depressive disorders				
Swedish-born with depressive disorders	2.78 (2.73, 2.84)	1.39 (1.36, 1.42)	15.55 (15.08, 16.03)	3.57 (3.45, 3.69)
Refugees with depressive disorders	5.80 (5.52, 6.08)	1.96 (1.87, 2.06)	27.43 (25.56, 29.43)	5.29 (4.92, 5.69)
Bipolar disorders				
Swedish-born with bipolar disorders	2.85 (2.71, 2.99)	1.38 (1.31, 1.45)	26.69 (25.29, 28.17)	4.88 (4.61, 5.16)
Refugees with bipolar disorders	4.95 (3.98, 6.15)	1.86 (1.50, 2.32)	36.08 (28.00, 46.48)	7.20 (5.58, 9.28)
Anxiety disorders				
Swedish-born with anxiety disorders	2.70 (2.64, 2.76)	1.41 (1.38, 1.44)	14.24 (13.79, 14.69)	3.86 (3.73, 4.00)
Refugees with anxiety disorders	5.81 (5.51, 6.13)	2.09 (1.98, 2.20)	22.11 (20.29, 24.09)	5.22 (4.78, 5.70)
Post-traumatic stress disorder (PTSD)				
Swedish-born with PTSD	2.90 (2.62, 3.20)	1.29 (1.16, 1.43)	26.59 (23.89, 29.6)	4.28 (3.84, 4.78)
Refugees with PTSD	6.25 (5.83, 6.70)	2.03 (1.89, 2.18)	33.40 (30.38, 36.72)	7.07 (6.42, 7.78)
Other stress-related disorders				
Swedish-born with other stress-related disorders	2.91 (2.83, 3.00)	1.41 (1.37, 1.45)	12.63 (12.08, 13.21)	3.07 (2.92, 3.21)
Refugees with other stress-related disorders	6.72 (6.35, 7.11)	2.25 (2.13, 2.38)	19.04 (17.17, 21.13)	4.76 (4.28, 5.28)
Other mental disorders				
Swedish-born with other mental disorders	3.52 (3.46, 3.57)	1.54 (1.52, 1.57)	15.75 (15.32, 16.18)	4.39 (4.25, 4.53)
Refugees with other mental disorders	6.02 (5.72, 6.33)	1.99 (1.89, 2.10)	22.87 (21.05, 24.84)	6.88 (6.32, 7.48)

Model 1 adjusted for sex, age, educational level, type of living area, family situation, labour market attachment, unemployment at baseline, sickness absence at baseline, somatic hospitalization or specialized outpatient care 2006–2009

*CI* confidence interval, *HR* hazard ratio, *Ref* reference category for each diagnostic group of mental disorders

The highest aHRs for long-term unemployment for refugees was seen for anxiety disorders (aHR 2.09), PTSD (aHR 2.03), and other stress-related disorders (aHR 2.25). For disability pension, the highest aHRs for refugees were present for bipolar disorders (aHR 7.20); PTSD: (aHR 7.07); and other mental disorders (aHR 6.88).

## Discussion

### Main findings

Patterns of labour market marginalization of refugees differed markedly from patterns among Swedish-born individuals and mental disorders had a differential impact on these outcome measures depending upon refugee status. Refugee status turned out to be a strong risk factor of long-term unemployment (HR 2.7), and neither mental disorders in general nor specific mental disorders did further add to this risk. In contrast, mental disorders clearly increased the subsequent risk of disability pension of both Swedish-born individuals (HR 4.0) and refugees (HR 6.1). In the absence of mental disorders, refugee status had a considerable impact on the risk of disability pension (HR 1.4). With regard to specific mental disorders, the pattern that mental disorders did not add to the risk of unemployment in refugees applied across the overall spectrum of mental disorders. For disability pension, presence of specific mental disorders generally showed higher risk estimates in refugees compared to Swedish-born, with the highest specific risks for bipolar disorder (HR 7.2) and PTSD (HR 7.1).

### Refugees and mental disorders

The prevalence of specific mental disorders was considerably higher among refugees compared to Swedish-born individuals. In particular, PTSD had a prevalence of 1.3% among refugees, compared to just 0.1% in Swedish-born. This difference is likely related to traumatic experiences before, during and after the migration process [11]; and/or to a higher likelihood of diagnosing PTSD among refugees. The 13-fold higher prevalence of PTSD among refugees compared to natives is consistent with a previous large systematic review of data from adult refugees resettled in Western countries: In this review, the prevalence of PTSD was estimated at 9%, and tenfold compared to native populations of the same age [11]. In comparison to that study, both natives and refugees had much lower prevalence rates of PTSD in the present analysis. This lower prevalence might derive from the fact that only data from inpatient and specialized outpatient healthcare, and no data from primary healthcare, were used to assess specific mental disorders in this study. Considerable differences in the prevalence between Swedish-borns and refugees were also present for other mental disorders, i.e. depressive disorders, with a prevalence of 3.0% in refugees compared to 1.9% in Swedish-borns, and anxiety disorders (2.4% vs. 1.9%). Overall, most specific mental disorders appear to be more prevalent in refugees than in Swedish-borns, and these differences appear strongest for stress-related disorders. This might be related to traumatic experiences before, during or after the migration process [11]. Only bipolar disorder was more prevalent among Swedish-borns compared to refugees (0.3% vs. 0.2%).

### Refugee status and disability pension and unemployment

The finding that refugee status per se contributed considerably to the risk of subsequent disability pension also after adjustment for socio-demographics is noteworthy. Disability pension is granted based on medical assessment, and refugee status per se should not be relevant for the granting of disability pension. The finding might derive from differences of symptoms and/or their presentation in refugees compared to Swedish-borns; residual confounding from mental disorders or poor mental health; a greater likelihood of physicians to grant disability pension to refugees; reduced work ability; or other factors such as discrimination.

With regard to long-term unemployment, the lower risk of refugees with mental disorders compared to refugees without mental disorders indicates that mental disorders do not contribute at all to their risk of marginalization in terms of unemployment. This is consistent with previous studies of non-Western migrants [9] which did not show any further increase in their risks of long-term unemployment beyond that of the migrant status. This finding suggests that refugees with mental disorders are more likely to enter pathways of marginalisation other than long-term unemployment, most importantly disability pension, and is consistent with the high baseline risk of unemployment in the group of refugees without mental disorders.

### Refugees with mental disorders and subsequent labour market marginalisation

The risk estimates for refugees were considerably higher in the present analysis as compared to the previously reported estimates of non-Western migrants, particularly regarding disability pension [9]. For example, non-Western migrants with mental disorders had a nearly fivefold risk of disability pension in an earlier analysis as compared to Swedish-borns, but the risk for refugees in the present analysis was more than sixfold the risk of Swedish-borns without mental disorders [9]. These differences might derive from differences in study populations and study design—in particular, the earlier study population was younger as compared to the current population, but also from differences between refugees and non-Western migrants. Per definition, refugees are individuals who “have been forced to flee their country of residence because of persecution, war or violence” [12], which means that the likelihood of traumatization, mental health problems, and reduced capacity to work is higher as compared to other migrant groups. In accordance with this, the prevalence of mental disorders, particularly PTSD, turned out to be higher in refugees as compared to natives, and was also higher as compared to non-Western migrants in a previous study [9].

### Specific mental disorders

With regard to disorder-specific risks of long-term unemployment and disability pension, there is a scarcity of research in the published literature. In particular, findings for refugees as presented in this paper have not been reported previously. A previous study found that the risk of disability pension in residents up to 41 years in Sweden was considerably increased for affective disorders (HR 8.0) and the group of neurotic/stress-related and somatoform diseases (HR 6.0). The corresponding HRs for long-term unemployment were 1.3 and 1.3, respectively [9]. In the present study, the effect sizes for refugees turned out to be higher for refugees than for Swedish-borns. The largest effects sizes for refugees were found for bipolar disorder and PTSD, which both were related to a sevenfold risk for disability pension as compared to Swedish-borns without mental disorders. Overall, the differences in risk for disability pension between disorders were small to moderate, and the adjustment for health-related and socio-demographic variables resulted in a clear reduction of the differences seen between specific disorders and between Swedish-borns and refugees.

It is noteworthy that the adjustment for sociodemographic, socio-economic, and health-related variables resulted in a strong reduction of crude estimates. This attenuation was mainly due to the effects of education and health-related variables at baseline.

The finding that there were no clear differences in risk estimates for specific mental disorders with regard to long-term unemployment for refuges with these disorders compared to refugees without mental disorders, speaks for the assumption that the diagnostic peculiarities related to functional impairment do not matter when it comes to unemployment. Unemployment is, unlike disability pension, not based on medical assessment. Refugees have a high risk of long-term unemployment even in the absence of mental disorders—and refugees as well as Swedish-borns with severe mental disorders are more likely to be covered by disability pension in the Swedish social insurance system.

### Strengths and limitations

A study strength is the use of high quality data from validated Swedish nationwide registers providing large study populations with low loss to follow-up and with vast opportunities to include covariates in the analyses [13–15]. The present study included over 210,000 refugees and additionally a large comparison group of Swedish-born individuals.

The study also has some limitations. Specific mental disorders were defined by in- or specialised outpatient care, which mostly reflects medically more serious cases since individuals treated in primary health care could not be included due to the lack of respective data. Treatment for disorders such as PTSD might often occur in primary health care settings, particularly for Swedish-born individuals, resulting in some bias. Although the analyses were adjusted for previous marginalisation, some remaining residual confounding due to the bidirectional pathways between labour market marginalisation and mental disorders cannot be ruled out [16]. Further, a considerable number of individuals had missing data on reason for settlement to Sweden (*n* = 262,579). Based on information from Statistics Sweden, for refugees who came to Sweden before 1986 this information seems sporadic. Later on, the reason for migration is mainly missing for migrants from the European Union, who are no refugees. Missing values on refugee status should, therefore, not bias the present results [17].

## Conclusions

The present findings highlight that refugees have a higher prevalence of mental disorders than Swedish-born individuals and are at high risk of labour market marginalization. Refugee status in itself seems to fully explain the increased risk of long-term unemployment, but also an important proportion of the increased risk for disability pension. Specific programs tailored to the increased risk of marginalization for refugees with stress-related disorders such as PTSD, but also depressive disorders, which are all considerably more prevalent in refugees need to be developed. These programs need to address traumatization that occurs before, during or after the migration process in refugees and which is known to increase the risk of stress-related disorders.
